# Verification of the folkloric and anecdotal antidiabetic effects of *Hypoxis hemerocallidea* (Fisch., C.A. Mey. & Avé-Lall) and isolated, β-sitosterol using early-stage type II spontaneous diabetic mutant BKS-*Lepr*^db^ mice

**DOI:** 10.1186/s12906-022-03640-y

**Published:** 2022-06-20

**Authors:** N. M. Mkolo, O. O. Olaokun, P. H. King, I. Janse van Rensburg, J. N. Eloff, V. Naidoo

**Affiliations:** 1 Department of Paraclinical Sciences, Section of Pharmacology, Faculty of Veterinary Sciences, University of Pretoria, Pretoria, South Africa; 2 Department of Biology, School of Science and Technology, Sefako Makgatho Health Science University, Medunsa, Pretoria, 0204 South Africa

**Keywords:** *H. Hemerocallidea*, β-Sitosterol, Pre-diabetic mutant BKS-Leprdb mice, Spontaneously pre-diabetic mice and antidiabetic

## Abstract

**Background:**

Previous studies in our laboratory in ex vivo assays have demonstrated *H. hemerocallidea* extract as potential antidiabetic agent through increased insulin release from pancreatic beta cells. Thus, for this study the early stage type II spontaneous diabetic mutant mice model was used to evaluate and determine the degree of the antidiabetic efficacy of *H*. *hemerocallidea*.

**Methods:**

Eight-weeks-old type II spontaneous pre-diabetic mutant BKS-Leprdb mice were fed with feed supplemented with either *H*. *hemerocallidea* extract, isolated compound (β-sitosterol) or chlorpropamide (positive control) for 4 weeks. The haematological parameters, clinical chemistry, glucose tolerance, feed intake, faecal output and body weights were measured.

**Results:**

The blood glucose concentrations of all the animals treated with plant extract, β-sitosterol compound and non-treated pre-diabetic animals did not return to baseline levels. Only the β-sitosterol treatment and positive control groups resulted in a respective small decrease of 5.8 and 5.2% in the mouse weights over the study period, with no significant changes (*p* > 0.05) in food intake. However, there was a general trend for decrease in faecal output for all the groups. Albumin, triglycerides, and total cholesterol levels in β-sitosterol and chlorpropamide-treated animals were lower, relative to untreated-animals. Animals fed with plant extract showed large amounts of internal fat. There were no significant changes (*p* > 0.05) in total serum protein, globulin, alanine aminotransferase, alkaline phosphatase, urea nitrogen and creatinine attributed to administration of treatments. In all groups, some animals showed lesions associated with cardiac puncture. Few animals except animals treated with plant extract, showed presence of a left-ventricular hypertrophic cardiomyopathy. The liver and kidneys for all groups appeared macroscopically normal and the thymuses were small (±2 mg). There were pathological signs in some of the animals particularly in myocardial fibres, renal tubular, glomerular, hepatocyte granularity and pancreas islets. However, there was no significance trend between the groups.

**Conclusion:**

Based on the results, none of the treatments could be considered highly effective for the management of type II pre-diabetes as sole therapeutic intervention.

**Supplementary Information:**

The online version contains supplementary material available at 10.1186/s12906-022-03640-y.

## Introduction

Diabetes mellitus is one of the most important non-communicable diseases in the world [1, 2]. Thus, numerous treatment options are available from allopathic remedies to complementary medicines based on more traditional methods of treatment [3]. Of the available herbal remedies, *Hypoxis hemerocallidea* is a commonly used remedy locally, in South Africa. It has been documented that the corm of *H. hemerocallidea* incorporates phytosterol glycosides, notably β-sitosterol, rooperol (the aglycone of a hypoxoside, which is a 4, 4-diglucoside) and some sterolins [4, 5]. Currently, the precise chemical compound in *H. hemerocallidea* corm that is accountable for the observed antidiabetic potential of the ‘African Potato’ aqueous extract is still not known. Different investigators have proven that a host of secondary plant metabolites with various chemical structures (e.g., oleanane triterpenoid glycosides, oleanolic acid and carrageenin) possess the latter properties in various experimental animal models [6–14]. In all the current models, the plant has been investigated using streptozocin (STZ) induced diabetes in mice and rats [15, 16]. The hallmark of these models is the induction of diabetes by inducing damage to the pancreatic β-cells. While these types of models are helpful to determine if a plant extract can decrease plasma glucose concentrations using a non-pancreatic method, it discriminates against those agents that enhance pancreatic activity [17–21]. The previous studies in our laboratory using ex vivo models have shown that *H. hemerocallidea* functions by increasing insulin released from pancreatic β-cells [22]. Therefore, it is assumed that the true antidiabetic effect of *H*. *hemerocallidea* ex vivo is yet to be established in in vivo models. Thus, for this study, the early stage type II spontaneous diabetic mutant mice model was used to evaluate and determine the degree of antidiabetic efficacy of *H*. *hemerocallidea*. Type II spontaneous diabetic mutant BKS-*Lepr*^db^ mice carry a spectrum of genetic susceptibilities for example, insulin resistance, hyperglycaemia, pancreatic beta cell atrophy, hyperlipidaemia, hypoinsulinemic comparable to that noticed in human type II diabetes because of a spontaneous mutation in the leptin receptor gene. Rise in plasma insulin starts at 10 to 14 days and elevated blood sugar at four to 8 weeks [17, 18, 23]. Type II spontaneous diabetic mutant BKS-*Lepr*^db^ mice are utilized to model phases I to III of type II diabetes [18, 19]. Diagnosis at an early stage and controlling blood sugar, blood pressure and cholesterol can prevent or delay the complications associated with diabetes [20], thus early stage of this model were used.

With the plant containing β-sitosterol (a known natural anti-cholesterol compound), it can be speculated that this plant sterols would also be able to alleviate the defects seen in plasma cholesterol associated with diabetes mellitus.

## Materials and method

### Compound and plant collection, preparation, and authentication

Corms of *H. hemerocallidea* (Fisch., C.A. Mey. & Avé-Lall), approximately 10–15 cm in diameter growing in the wild habitat, were collected in Ga-Rankuwa, North-West province of South Africa. The identity and authentication of the plant was confirmed by a taxonomist at the National Herbarium of the South African National Biodiversity Institute in Pretoria where sample specimen was deposited and voucher specimens assigned (NR 201). Corms were cut into smaller pieces and were dried at room temperature before being ground into a powder. Corm powder (650 g) was extracted with 5.0 l of methanol on a Labotec shaker for 24 hours. The resulting extract was filtered using Whatman No. 1 filter paper to remove plant debris, and the filtrate dried under a stream of air at room temperature. This method was repeated to yield 250 g of the plant extract for further use.

*Hypoxis hemerocallidea* crude methanol extract was fractionated by solvent-solvent fractionation using the method of the National Cancer Institute in the USA [24] as cited and applied by Martini and Eloff [25]. This afforded five fractions (n-hexane, chloroform, water in methanol, butanol and water fractions) of the extract. The plant extract and fractions were separated by TLC (thin layer chromatography) using three solvent systems: ethyl acetate: methanol: water (E: M: W) (10:1, 35:1 v/v), chloroform: ethyl acetate: formic acid (CEF) (10:8:2) and ethyl acetate: butanone: water: formic acid (EBWF) (5:3:1:1). The chromatograms were subsequently sprayed with DPPH (1,1-Diphenyl-2-picrylhydrazyl) and vanillin sulphuric acid (1% solution of vanillin in concentrated sulfuric acid). Appendix A shows the TLC chromatograms with different compounds being separated.

The 100% pure β-sitosterol compound was obtained commercially from Sigma. The structure of the β-sitosterol compound was determined by one dimensioned (^1^H and ^13^C) and two-dimensional Nuclear magnetic resonance (NMR) spectroscopy, for authentication purposes. ^1^H NMR spectra were measured at 399 MHz (MHz) and the ^13^C NMR were measured at 100 MHz. All NMR experiments were conducted at a constant temperature of 26 °C with a Varian 400 MHz spectrometer. Chemical shifts (δ) were measured in parts per millions (ppm) from internal standard tetramethylsilane (TMS). The quantitative analysis results of β-sitosterol compound are presented in Appendix B.

### Animal material, caging and care

The early-stage type II diabetic spontaneous mutant male mice (BKS.Cg-*m+/+Lepr*^db^/BomTac (db/db); BKS background; 8 weeks; 29.0–42.0 g; *N* = 45), were housed individually in type II individually ventilated cages (IVC) from Tecniplast in rooms maintained at 22 ± 2 °C, 40–60% relative humidity with a 12 h light-dark cycle. The mice were fed irradiated standard rodent chow pellets (Epol, South Africa), reverse osmosis water supplemented ad libitum and housed on cloth bedding material (Agrebe™). Cages were weekly changed. The animals were obtained from the central animal facility of Taconic Biosciences Inc., USA, and transferred to Denmark (Vet import permit from Denmark) and then shipped further to South Africa. Autoclaved toilet rolls, egg containers, tissues and wooden sticks were provided as environmental enrichment. The cloth bedding was to allow for faecal collection without having to place the animals into metabolic cages. Prior to the study, approval of the protocols with number V113–17 was obtained from the University of Pretoria, Animal Ethical Committee and the experiments were conducted in accordance to the standard guidelines (CPCSEA, OECD guidelines no 420). In additional all methods are reported according to the ARRIVE guidelines for the reporting of animal experiments.

To allow acclimatisation, animals were maintained on the irradiated standard rodent chow pellets (Epol diet made from the ingredient of protein 180 g/kg, moisture 120 g/kg, fat 25 g/kg, fibre 60 g/kg, calcium 18 g/kg and phosphorus 8 g/kg) and water for 1 week. The initial body weights of animals were measured, then divided randomly into four groups (*n* = 11) for food treatment; Group A: Epol diet; Group B: Epol diet and β-sitosterol; Group C: Epol diet and plant methanol extract and Group D: Epol diet and positive control chlorpropamide, using EXCEL computer software. The experimental Epol pellets were milled for the control and treatment groups, then the milled Epol diet of the treatment groups was supplemented with test material. The food was prepared for each treatment separately as plant extract (250 mg) or β-sitosterol (compound) (30 mg) or chlorpropamide (30 mg) added to 1 kg of Epol standard rodent chow according to CPCSEA, OECD guidelines no 420 (Fixed Dose Procedure). Animals were maintained on their medicated feed for 4 weeks.

### Glucose tolerance test

After a week on treatment, blood glucose levels were measured using a hand-held glucose meter (OneTouch Ultra, United States). Emla cream (lidocaine 2.5% and prilocaine 2.5%) was applied 2 hours before tail prick. For the glucose tolerance test (ipGTT), a prior overnight (16 hours) fasting was done and on the following day after baseline blood glucose levels were measured, a sterile solution (dextrose 50%) at 2 g of glucose/kg of body weight was injected intraperitoneally (ip). Glucose levels were subsequently evaluated at 30, 60, 90, and 120 min. The entire procedure was repeated after a week and then again after another 2-week periods.

### Feeds intake, faecal output, and animal weights

Feed intake on a Monday and Friday every week was recorded. The amount of wasted feed at the bottom of the cage was also measured to get a proper indication of the feed intake. The faecal output and animal weights were also measured on Monday and Friday of every week.

### Clinical pathology

The animals were terminally anesthetized by isoflurane insufflation in a saturated bell jar and subjected to cardiac puncture. Blood samples were submitted on an automated analyser **(**IDEXX ProCyte Dx). The haematological parameters measured were red blood cell count (RBC), white blood cell count (WBC), haemoglobin (Hb), haematocrit, mean corpuscular volume (MCV), neutrophils, eosinophils, basophiles, lymphocytes, monocytes, and platelets count (PLT). Serum chemistry parameters included alkaline phosphatase activity (ALP), alanine aminotransferase (ALT), urea, plasma glucose, creatinine, total cholesterol, globulin, albumin, and triglycerides.

### Terminal pathology

During necropsy, gross morphological findings were recorded and specimens were collected from each individual in 10% buffered formalin. The following organ specimens were collected for histological examination: heart, liver, kidney, adrenal gland, pancreas, and peripheral nerves in the anterior mesenteric region and/or hind leg (medial mid-thigh). After fixation in 10% buffered formalin, the organs were cut according to standard operating procedure (IdexxSA-AP-SOP-26) using automated tissue processor (IdexxSA-AP-SOP-27). Following tissue processing, sections were cut of 6 μm (IdexxSA-AP-SOP-30), and the slides produced stained in an automated Haematoxylin and Eosin tissue stainer (IdexxSA AP-SOP-205). In addition to standard histopathological evaluation, the pancreatic islet size and diameter were quantified between the groups using the NIS-elements AR Imaging (Nikon, Japan) software program. All cuts were captured at 100x and 400x magnification, using 10x and 40x objectives. Morphometric analyses were performed to calculate average islet diameter using eighty-eight stained sections (8 from each mouse) per group of A, C or D and eighty stained sections for group B. The total of islets per 11,700 μm^2^ fixed square area was calculated. Each group measurements were averaged. A total of 1894, 1466, 1099 and 1649 islets from all mice fed with β-sitosterol, Epol diet only, plant extract and chlorpropamide were measured, respectively. Normal islets of Langerhans were conceded to be around 50–500 μm in diameter, while less than 50 μm in diameter were considered to be small and more than 500 μm in diameter were considered to be enlarged [26].

### Data analysis

All data analyses were done using Graph Pad Prism software. However, the sample size was estimated per group using G*Power 3 statistical software and the sample of 45 had sufficient power of 0.80. Sample size of 11 mice was used per group and all values were expressed as mean ± standard deviation (mean ± SD). Tukey’s test was used to determine the significance of the difference among samples (body weights, food intake of the mice and blood glucose concentration) treatment per group (per time point). Chi-square test for histological findings were used to determine whether there was a significant (*p* < 0.05) difference between the expected frequencies and the observed frequencies in categories. This was achieved by categorising the histological changes of each mouse heart, liver, kidney, adrenal gland and pancreas into normal, mild changes, moderate changes and server changes. The proposed comparison of efficacy between β-sitosterol compound, positive control chlorpropamide and plant extract is based on changes in fasting blood glucose levels from baseline for 4 weeks period. The fasting blood glucose levels (16 hrs) of around 150–300 mg·dL^− 1^ (8.3–16.7 mM) was considered as primary endpoint for 4 weeks period.

## Results

### Monitoring of experimental animals

Careful monitoring of experimental animals was done to establish when an animal has reached the humane endpoint in the study. The appearances and behaviour of each mouse during the antidiabetic study were observed (Table 1). However, one animal died after 1 week on compound treatment (B group). In addition, dark-coloured stools were observed in animals treated with a plant extract (C group). There was a decrease in the body mass of the compound or positive control-treated groups (B and D groups) compared to the Epol diet (control)-treated or plant extract-treated groups (A and C groups). However, these animals recovered within 14 days and gained weight. Moreover, there were no observed behavioural, autonomic, neurological, physical changes like motor activity alertness, convulsions, restlessness, lacrimation and coma for 4 weeks study period.

**Table 1:** General appearance and behaviour of the mice during the antidiabetic study

	GROUP A		GROUP B		GROUP C		GROUP D	
	On day 3	On day 14 until day 44	On day 3	On day 14 until day 44	On day 3	On day 14 until day 44	On day 3	On 14 day until day 44
**Skin and fur**	NT***	All Normal	NT***	Normal	NT***	All Normal	NT***	Normal
**Eyes**	Normal		NT***	Normal	Normal		NT***	Normal
**Behavioural pattern**	Normal		Weak and shivering	Normal and active	Normal		Weak and shivering	Normal and active
**Stool-colour**	Normal		Mixed with wasted food	Normal	Darker stools		Mixed with wasted food	Normal
**Sleep**	Normal		NT***	Normal	Normal		NT***	Normal
**Diarrhoea**	Not observed		Not observed	Not observed	Not observed		Not observed	Not observed

Group A: Standard pellet diet and water; Group B: Standard pellet diet, water, and compound; Group C: Standard pellet diet, water, and plant extract; Group D: Standard pellet diet, water, and positive control. N.B: the animal food (25 g) was mixed with natural apple essence 30 ul (sugar free flavouring) to make the food more palatable. Since it was observed that groups B and D animals did not eat for the first 3 days post-added natural apple essence was supplemented. Key: NT*** not normal

### Blood glucose concentrations

**Fig. 1:**
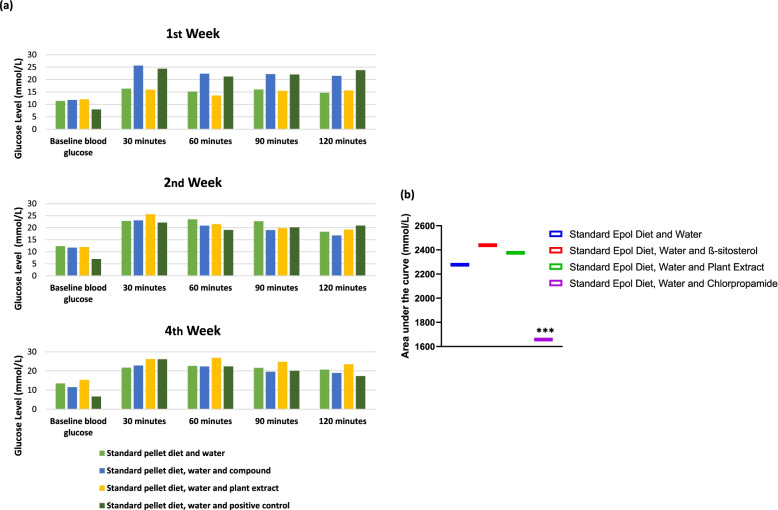
Blood glucose concentrations (mmol/L) of different tested treatments on spontaneous pre-diabetic mutant BKS-*Lepr*^db^ mice for 4 weeks period of treatments. **a** The animals treated with plant extract, β-sitosterol compound and non-treated animals showed no significant reduction of blood glucose levels (mmol/L) after ip-glucose solution administration evaluated at 30, 60, 90, and 120 mins for 4 weeks of treatment. **b** Demonstration of the area under the curve (AUC) after 4 weeks of treatment, *** *p ≤ 0.05*, significant difference of animals treated with positive control when compared with the plant extract, β-sitosterol compound and non-treated animals

**Fig. 2:**
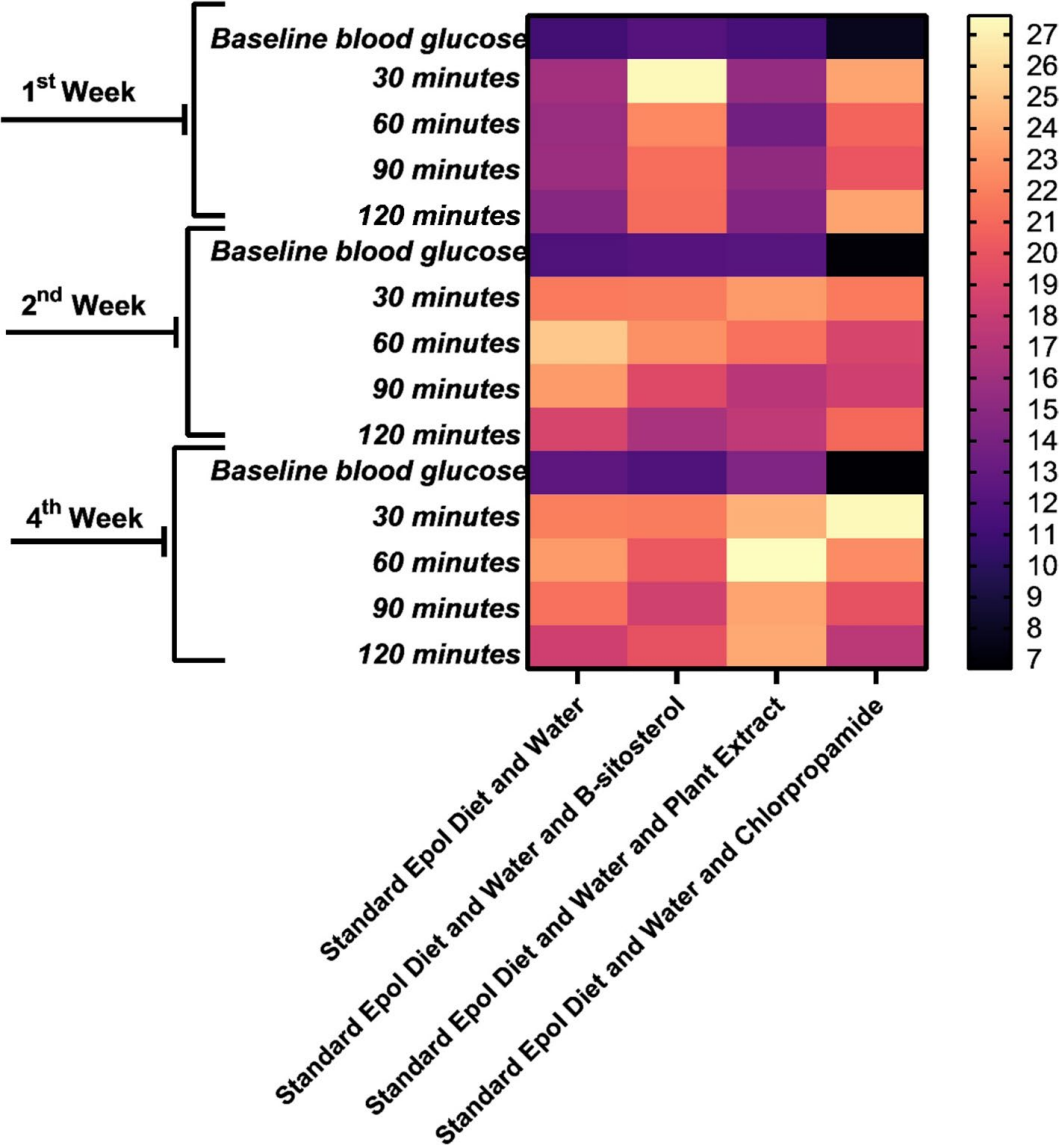
Heatmap clustering plot of blood glucose concentrations (mmol/L) of spontaneous pre-diabetic mutant BKS-*Lepr*^db^ mice after standard pellet diet, β-sitosterol compound, plant extract or positive control chlorpropamide administration for 4 weeks

The results of the ipGTT performed at week 4 are presented in Figs. 1 and 2. All the animals were prediabetic at the start of treatment, with no significant changes (*p* > 0.05) evident over time (4 weeks period) for the β-sitosterol compound, while the plant extract and control had continuously increasing blood glucose level. However, the positive control showed significant reduction (*p* < 0.05) of blood glucose level, evident after 2 weeks (Fig. 2). After intraperitoneally (ip) injecting a solution of glucose, the blood glucose concentrations of all the animals treated with plant extract, β-sitosterol compound and non-treated prediabetic animals (except those treated with positive control), did not return to baseline levels at the end of 120 minutes after 1, 2 and 4 weeks of treatment (Fig. 1A). The area under the curves (AUC) of the animals which were plant extract and β-sitosterol compound treated showed no significant difference (*p* > 0.05) compared with non-treated animals. However, the AUCs of the animals treated with positive control after 4 weeks showed significant difference (*p* < 0.05) when compared with animals treated with plant extract, β-sitosterol compound and non-treated animals (Fig. 1B). This further designate that the plant extract and β-sitosterol compound does not reduce the blood glucose levels of the tested mice.

### Body and faecal weight and food intake

**Fig. 3:**
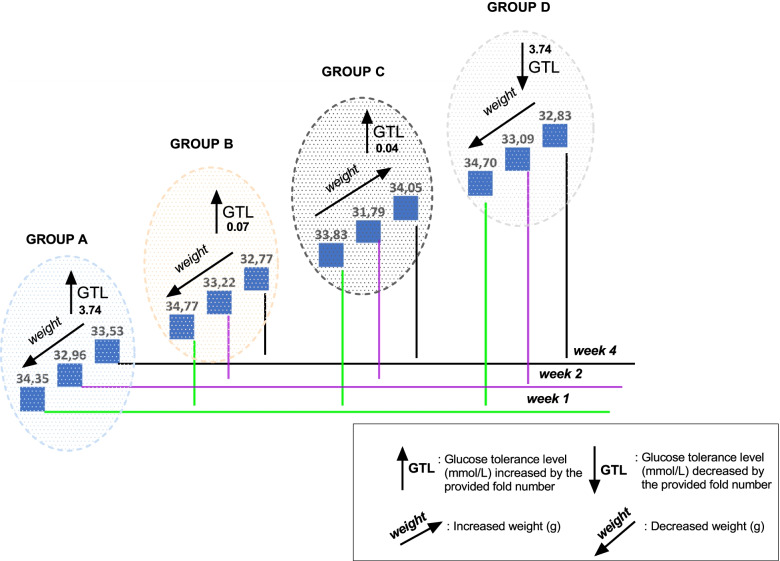
Illustration of the relationship between the blood glucose concentrations (mmol/L) and body weights (g) of different tested treatments and untreated spontaneous pre-diabetic mutant BKS-*Lepr*^db^ mice for 4 weeks period. A general trend of the increase/decrease of blood glucose concentrations (mmol/L) and weights (g) of mice. Key: Group A: Epol diet and water; Group B: Epol diet, water and β-sitosterol; Group C: Epol diet, water and plant extract and Group D: Epol diet, water, and positive control

The body weight of the mice decreased when treated with β-sitosterol compound, chlorpropamide and non-treated animals. A decrease difference was not significant (*p* > 0.05) between the treatments (groups B, C or D) and the control (group A), which was evident as slight weight loss of 2.4, 5.8 and 5.4% for group A, B and D, respectively. Evident as a concurrent decrease in blood glucose concentrations was seen, between the blood glucose concentrations (mmol/l) and the weights (g) of the animals after 1, 2 and 4 weeks of treatments (groups D) (Fig. 3).

**Fig. 4:**
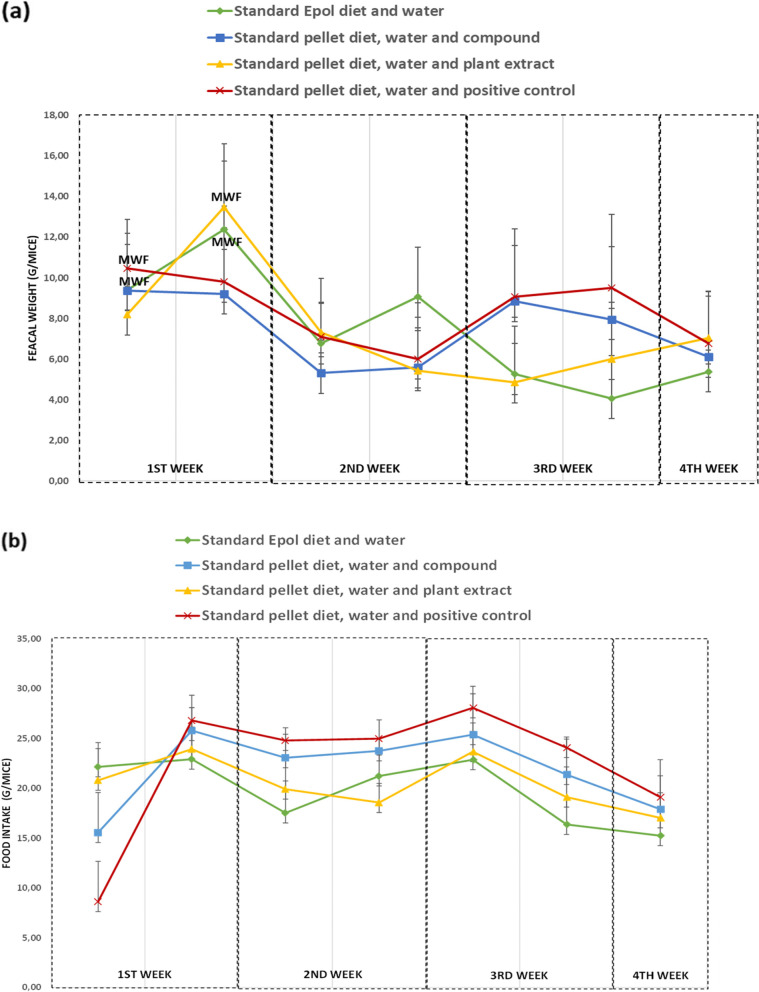
Effect of food (treatment and control) administration on the faecal weights of spontaneous pre-diabetic mutant BKS-*Lepr*^db^ mice for 4 weeks period. **a** A general trend of decrease in faecal output of spontaneous pre-diabetic mutant BKS-*Lepr*^db^ mice after 4 weeks administration of standard pellet diet, β-sitosterol compound, plant extract or positive control chlorpropamide. **b** A general trend of decrease in food intake of spontaneous pre-diabetic mutant BKS-*Lepr*^db^ mice after 4 weeks administration of β-sitosterol compound and positive control chlorpropamide. Key: MWF = Faeces mostly mixed with food. Data are presented as mean ± SD (*n* = 11)

Food intake did decrease after 4 weeks of β-sitosterol compound and chlorpropamide treatment (Fig. 4B). There was no significant difference between (*p* > 0.05) the treatments (groups B, C or D) and the control (group A) after 4 weeks of treatments. There was a general trend for decrease in faecal output for all the groups (Fig. 4A).

### Clinical pathology

The haematological parameters obtained for the study are presented in Table 2 and clinical chemistry in Table 3. There were no changes in the haematology evaluations attributable to administration of treatments. Albumin, triglycerides, and total cholesterol levels in β-sitosterol and chlorpropamide-treated animals were lower, relative to untreated-animals (group A). Treatment of animals with doses of plant extract, β-sitosterol and chlorpropamide resulted in non-significant changes in total serum protein, globulin, alanine aminotransferase, alkaline phosphatase, urea nitrogen and creatinine. In untreated and β-sitosterol and plant extract-treated animals, there were increases in glucose levels of 21.63 ± 0.21 mmol/l, 19.91 ± 2.81 mmol/l and 17.35 ± 1.18 mmol/l, respectively. In chlorpropamide-treated animals the glucose level was 10,20 ± 0,62 mmol/l.

**Table 2:** Haematological parameters of mice

Full blood count Parameters	Group A	Group B	Group C	Group D	Reference range (Ref**)
Haemoglobin (g/L)	134,9 ± 10.38	136,125 ± 5,10	131,60 ± 5,10	134,91 ± 8,57	135–163
Red Cell Count (x10Ʌ12/L)	9,46 ± 0,78	9,19 ± 0,44	9,33 ± 0,44	9,41 ± 0,56	9.82–10.65
Haematocrit (L/L)	0,44 ± 0,04	0,44 ± 0,02	0,42 ± 0,02	0,44 ± 0,03	0,40-0,46
Mean corpuscular volume (fL)	46,67 ± 0,50	46,2 ± 1,04	45,16 ± 1,04	46,29 ± 0,51	43,7-45,6
Mean corpuscular haemoglobin (pg)	14,25 ± 0,14	14,425 ± 0,17	14,10 ± 0,17	14,35 ± 0,18	14,8–16.0
Mean corpuscular haemoglobin concentration (g/dL)	30,56 ± 0,54	31,19 ± 0,73	31,21 ± 0,73	31,00 ± 0,40	33,3-34,9
Red cell distribution width (%)	13,03 ± 0,70	12,2 ± 0,33	12,86 ± 0,33	12,24 ± 0,35	11.5–14.5
White cell count (x10Ʌ9/L)	8207 ± 1,78	8,25 ± 1,30	6,50 ± 1,30	7,89 ± 1,07	7,80-15,61
Segmented neutrophil (x10Ʌ9/L)	1706 ± 0,71	1,99 ± 0,67	1,39 ± 0,67	1,64 ± 0,65	1,50-4,02
Band neutrophil (x10Ʌ9/L)	0,00 ± 0,00	0,00 ± 0,00	0,00 ± 0,00	0,05 ± 0,08	0–1
Lymphocyte (x10Ʌ9/L)	5,36 ± 1,77	5,57 ± 0,90	4,26 ± 0,90	5,48 ± 1,37	6,63-12,46
Monocyte (x10Ʌ9/L)	0,51 ± 0,27	0,475 ± 0,16	0,32 ± 0,16	0,32 ± 0,13	0–0,45
Eosinophil (x10Ʌ9/L)	0,58 ± 0,22	0,215 ± 0,17	0,52 ± 0,17	0,40 ± 0,27	0–0,45
Basophil (x10Ʌ9/L)	0,06 ± 0,10	0 ± 0,04	0,01 ± 0,04	0,01 ± 0,02	0–0,09
Platelet count	1067,8 ± HSD	993,125 ± HSD	1195,90 ± HSD	957,55 ± HSD	862–1611

HSD = Very high standard deviation difference > 100; Ref** Serfilippi et al. [27]

**Table 3:** Serum chemistry parameters of mice

Full blood count Parameters	Group A	Group B	Group C	Group D	Reference Range (Ref**)
Total serum protein (g/l)	45,83 ± 1,78	44,91 ± 1,85	44,02 ± 2,24	45,93 ± 1,88	44–58
Albumin (g/l)	22,06 ± 1,89	20,42 ± 2,80	22,15 ± 4,61	18,52 ± 2,77	26–38
Globulin (g/l)	23,75 ± 1,73	25,49 ± 2,40	26,87 ± 2,78	27,40 ± 1,98	17–22
Alanine aminotransferase(U/l)	26,78 ± 5,03	28,76 ± 8,78	26,09 ± 7,42	26,55 ± 3,73	31–57
Alkaline phosphatase (U/l)	73,27 ± 17,36	66,80 ± 13,75	68,64 ± 14,16	69,91 ± 12,76	55–100
Urea nitrogen (mmol/l)	7,00 ± 0,53	5,94 ± 0,62	5,94 ± 0,85	5,80 ± 0,90	1,3–7
Creatinine (mmol/l)	< 18 ± 0,00	< 20,00 ± 1,41	< 18 ± 0,00	< 18 ± 0.00	0,016-0,02
Cholesterol (mmol/l)	4,23 ± 0,16	3,10 ± 1,98	4,19 ± 0,20	2,56 ± 0,19	4,05-5,49
Triglyceride (mmol/l)	1,31 ± 0,21	1,11 ± 0,44	1,36 ± 0,21	1,08 ± 0,29	2,05-5,4
Glucose (mmol/l)	21,63 ± 0,21	19,91 ± 2,81	17,35 ± 1,18	10,20 ± 0,62	4–7

Ref**Serfilippi et al. [27]

### Pathological changes

Necropsy findings are presented in Table 4. The animals were all in good condition, although with various amounts of internal body fat present. Animals fed with Epol diet only, β-sitosterol and chlorpropamide showed moderate amounts of internal body fat but animals fed with plant extract showed large amounts of internal fat. The histopathological descriptions of each mouse are presented in Table 5. While changes were observed in the heart, kidneys and liver, no specific relationship to any of the treatment groups were present, with the exception of mice treated with the plant extract with most animals in this group showing a single small aggregate of lymphocytes and neutrophils which were present in the hepatic parenchyma.

**Table 4:**
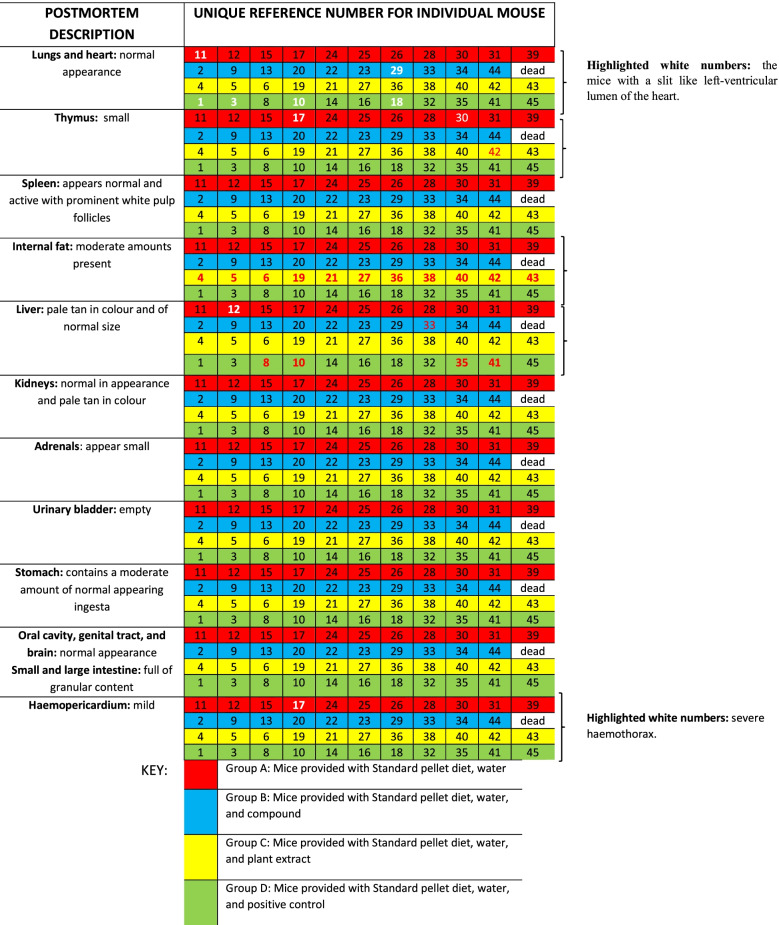
Necropsy morphological findings

**Table 5:**
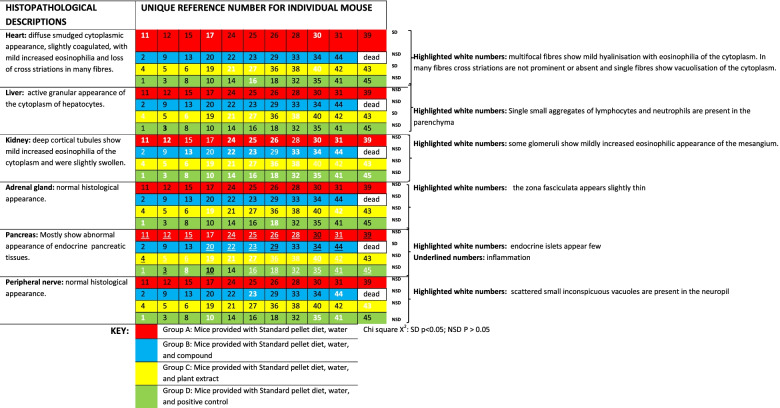
Histological descriptions of each mouse

**Fig. 5:**
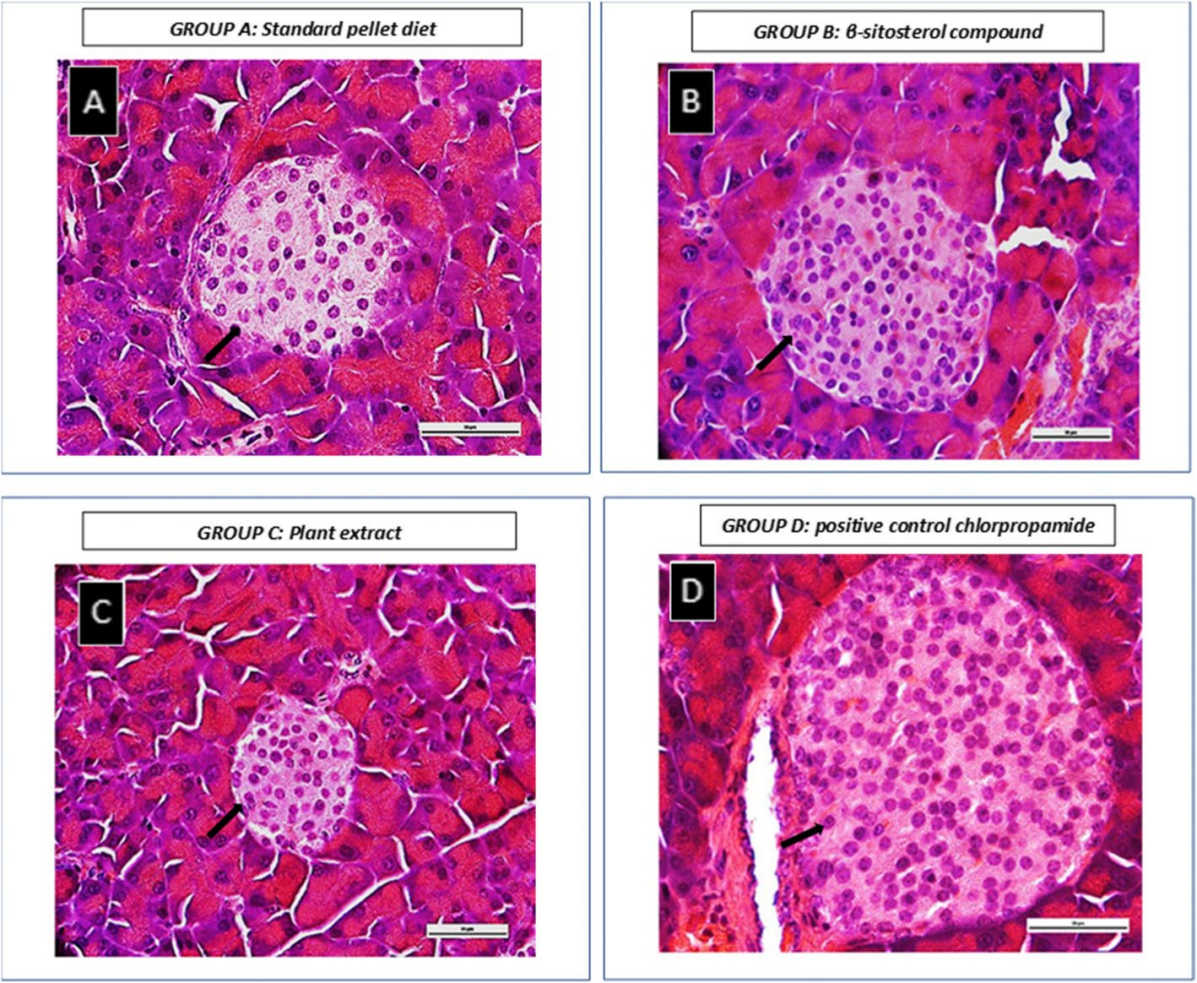
Representative images of the effect of food (treatment and control) administration in the islets of Langerhans in pancreas of spontaneous pre-diabetic mutant BKS-*Lepr*^db^ mice for 4 weeks showing different distribution pattern of endocrine cells. **Group A and C:** Pancreatic sections from animals fed with Epol diet only and plant extract mainly showed endocrine cells that are scant. **Group B and D:** Pancreatic sections from animals fed with β-sitosterol and chlorpropamide contained several endocrine cells. (Haematoxylin-eosin staining, 400× and 100x). Scale bars, 50 μm. Key: Black arrow- Endocrine cells

Few animals in all groups had some pancreatic tissue abnormalities described as normal exocrine and endocrine pancreatic tissues mostly, with one or more endocrine islets surrounded by interstitial lymphocytic aggregates with few increased septal fibroblasts, which was suggestive of inflammation (Table 5). There were also variations in the distribution pattern of endocrine islets of Langerhans. Pancreatic sections from animals fed with Epol diet only and plant extract mainly showed endocrine islets of Langerhans that are scant (Fig. 5A and C). Most sections from animals fed with β-sitosterol and chlorpropamide contained several endocrine islets of Langerhans, while other sections did not clearly contain any (Fig. 5B and D). These numerous endocrine islets of Langerhans were in some cases surrounded by interstitial lymphocytic aggregates and few increased septal fibroblasts. The endocrine islets of Langerhans of animals fed with Epol diet only, β-sitosterol and chlorpropamide appeared coalescing in some areas and slightly expanded in size when compared with animals fed with plant extract (Fig. 7).

**Fig. 6:**
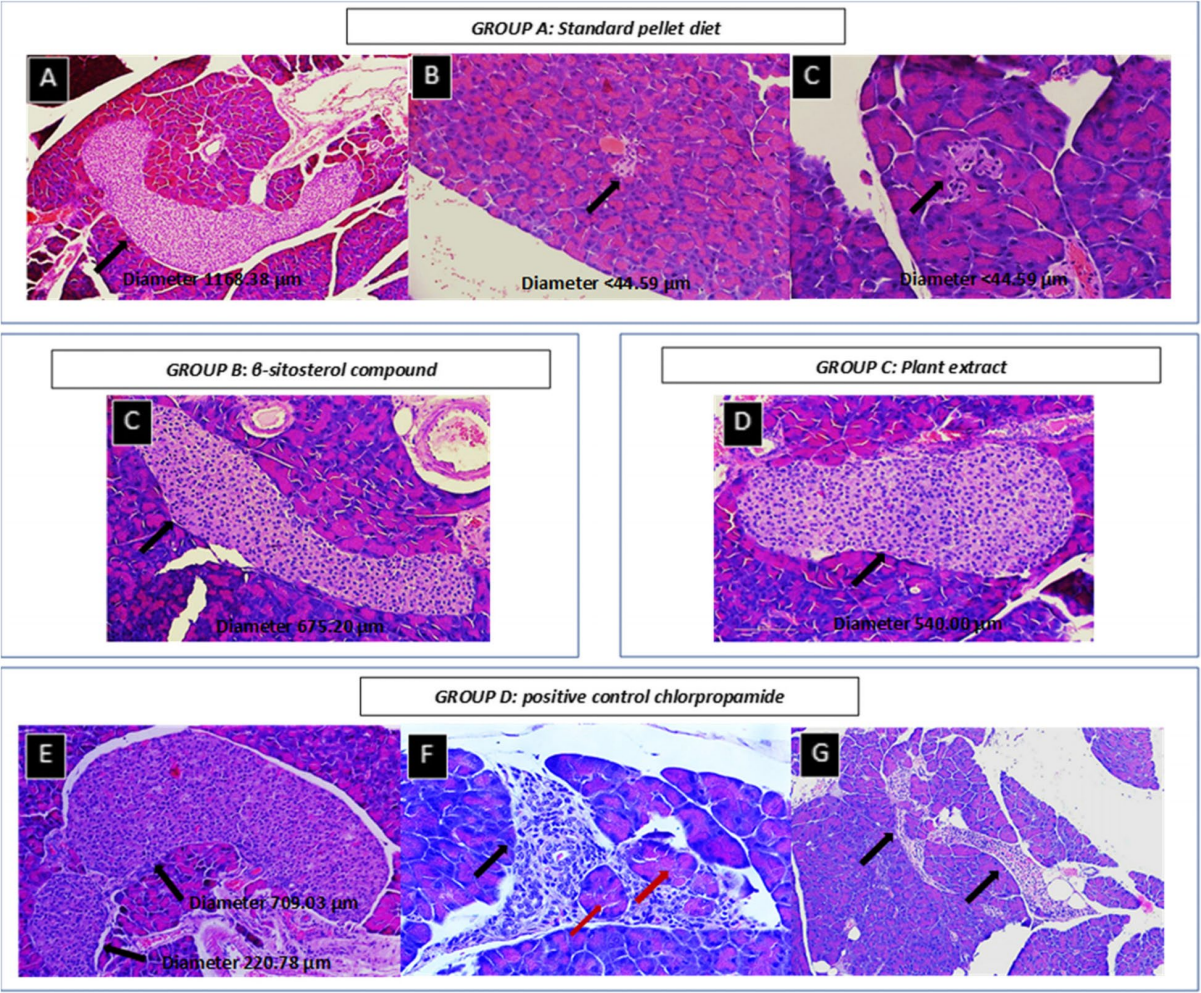
A photomicrograph example of a pancreatic section of spontaneous pre-diabetic mutant BKS-*Lepr*^db^ mice after standard pellet diet, β-sitosterol compound, plant extract or positive control chlorpropamide administration for 4 weeks. The islets are variably enlarged due to hyperplasia of cells and fusion of multiple islets. Pancreatic islet cell hyperplasia is characterized by enlargement of islets (up to 500 μm in diameter). While other islets are variably small due to degeneration of cells. Pancreatic islet cell atrophy is characterized by decrease of islets (less than 50 μm in diameter). **Group A:** Consist of pancreatic islet cell hyperplasia (Slide A; diameter 1168.38 μm) and atrophy (Slide B and C; diameter <44.59 μm) in spontaneous pre-diabetic mutant BKS-*Lepr*^db^ mice fed with standard pellet diet for 4 weeks. **Group B and C:** Consist of pancreatic islet cell hyperplasia (Slide D; diameter 675.20 μm) and (Slide E; diameter 540.00 μm) in spontaneous pre-diabetic mutant BKS-*Lepr*^db^ mice fed with β-sitosterol compound or plant extract for 4 weeks. **Group D:** Consist of pancreatic islet cell hyperplasia (Slide F; large islet diameter 709.03 μm, connected to small islet diameter 220.78 μm) and few exocrine acini trapped near the periphery (Slide G) in spontaneous pre-diabetic mutant BKS-*Lepr*^db^ mice fed with positive control chlorpropamide for 4 weeks. (Haematoxylin-eosin staining, 400× and 100x). Scale bars, 50 μm. Key: Black arrow- Islets of Langerhans; Red arrow- Exocrine acini

**Fig. 7:**
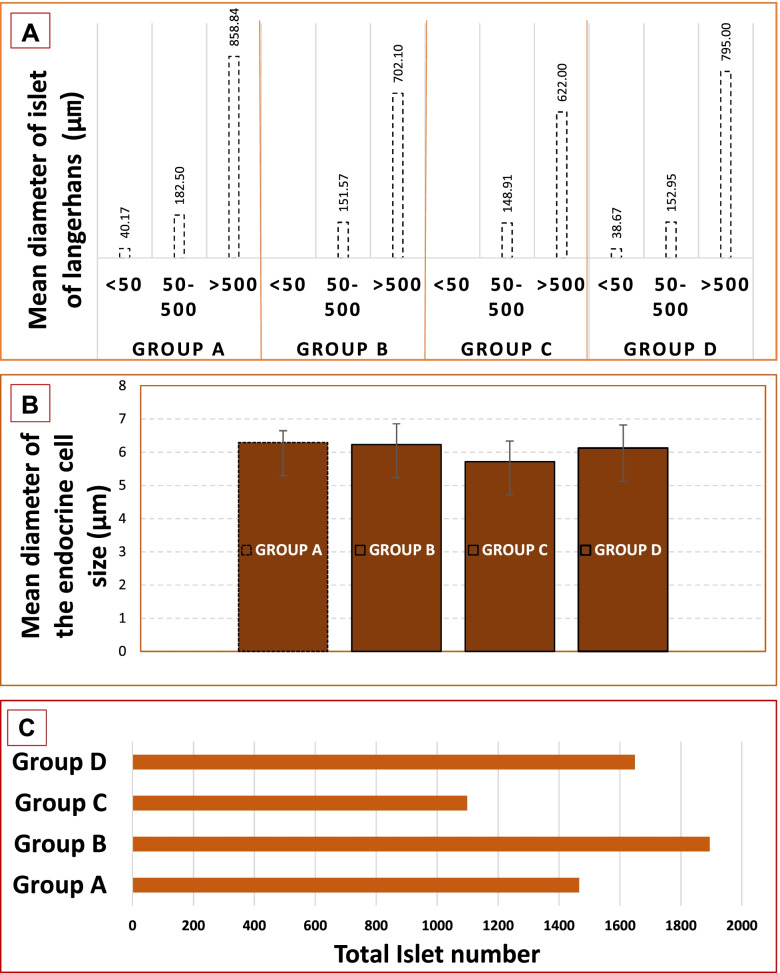
Illustrates an average diameter (μm) of islet of Langerhans and endocrine cells size distribution obtained from all 8 weeks old spontaneous pre-diabetic mutant BKS-Leprdb mice. **A** Variation in size of islets Langerhans which is not limited to ~ 500 μm in diameter, (**B**). Variation in size of endocrine cells which is not limited to ~ 10 μm in diameter and (**C**). β-sitosterol treated group had an increase in total islet number compared to Epol diet, plant extract and chlorpropamide treated groups. Key: Group A: Epol diet and water; Group B: Epol diet, water and β-sitosterol; Group C: Epol diet, water and plant extract and Group D: Epol diet, water, and positive control

Histopathologic changes of islets for animals fed with Epol diet only and chlorpropamide (Fig. 6), showed some islets which are hyperplasia and others which are atrophy (Fig. 7). In some cases, animals fed with β-sitosterol and plant extract showed hyperplasia (Figs. 6 and 7). Morphometric analysis revealed that β-sitosterol group had an increase in total islet number compared to Epol diet group, no differences were observed for plant extract and chlorpropamide groups (Fig. 7).

## Discussion

In this study, efficacy was determined using a spontaneous mouse model of diabetes through the use of the BKS.Cg-m+/+Leprdb/BomTac (db/db) mouse strain. The specific mouse model was selected as the leptin receptor gene mutation in BKS-*Lepr*^db^ mice increases susceptibility to type 2 diabetes [20]. Despite the model providing the necessary pathology for the proper study of efficacy, two specific modifications to the model need to be highlighted. Firstly, the study made use of males only, since they generally show abnormalities from as early as 8 weeks of age [23, 28], making them a better indicator of treatment responses in earlier stage of type II diabetes. Despite the benefits of using makes, the following study cannot accommodate for hormonal influences that may result from sex-related differences. The second consideration was the overnight (16 hours) fasting implemented before the glucose tolerance test (ipGTT). While the risks of such long fasts could result in significant weight loss and is non-consistent with shorter periods of fast recommended for people before a GTT is undertaken, the longer period was selected based on previous studies which showed that mice fasted for 16 hours had significantly (*p* < 0.05) lower plasma glucose concentrations than mice fasted for 4 h [29–31], thereby narrowing individual difference for the GTT i.e. sixteen hours fasting has the benefit of lowering irregularity in baseline blood glucose. Nonetheless, the animals were always closely monitored for weight loss immediately after the fasting period to ensure compliance with ethical standards. When weight loss was noted, it was never more than 1% of the animals’ weight from the day before. This would therefore indicate that the results obtained were not due to the animals losing excessive weight.

The plant extract and control led to an increased in blood glucose level despite treatment used, for 4 weeks study period. The results from this study thus does not support previous studies that have indicated that *H. hemerocallidea* corm extract has clear antidiabetic properties [15, 32]. This may be due to the different model used. As mentioned in the foregoing paragraphs, the animals used in this study were type II spontaneous diabetic mutant BKS-*Lepr*^db^ mice and not induced through chemical means, where the other studies used STZ-induced diabetes in mice and/or rats [15, 32]. *H. hemerocallidea* corm is known to have antioxidant activity [33, 34], the findings from previous studies may thus only show the ability to mitigate in diabetes developing in the presence of oxidative stress as opposed to treating disease once present.

The results of this study also showed no change in glucose concentrations over time (4 weeks period) for β-sitosterol, while the control showed continuous increase. This would support efficacy, as β-sitosterol mitigated to an extent the increase in blood glucose concentrations which is likely an indication of disease progression in the control and extract treated group. This trend for stabilised blood glucose was also present for the positive control. In the study by Sanjay et al. [35], β-sitosterol has antidiabetic activity in treated mice, in addition to reversing the weight loss. The reason for the lack of response in the extract group may be because the concentration of β-sitosterol was too low therein. This would support a study [4] which speculated that β-sitosterol is unlikely to account solely for antidiabetic activities of the corm’s aqueous extract used for in vivo study, since the compound is usually more abundant in alcoholic extracts, rather than in aqueous extracts of the corm. Moreover, this can be due to the fact that the absolute bioavailability of β- sitosterol compound upon oral administration is about 9% [36].

The *H. hemerocallidea* corm extract failed to reverse the weight gain of mice, also with no decrease in food intake and faecal output, throughout the experiment. In contrast, the other two groups treated with β-sitosterol and positive control of chlorpropamide with no significant difference (*p* > 0.05), showed some weight loss together with a reduction in the 120 min post-exposure blood glucose concentrations. This finding is likely explained by a late 2017 studies which have suggested that type II diabetes can be reversed, not through original pharmaceutical treatments, but through firm adherence to certain dietary interventions of low carbohydrate diet with 850 total daily calories for humans [37]. The Epol diet, β-sitosterol and chlorpropamide groups led to a slight decrease of 2.3, 5.8 and 5.2% in animal weights, respectively. Specific mechanism behind this slight weight reduction of these groups is unknown, however it could be due to changes in food consumption, inhibition of intestinal lipid absorption [38–41], an increase in the expenditure of energy [42], stimulation of lipid oxidation [42, 43] or fasting of the animals [31]. The animals showed no change in food intake regardless of the treatment used. However, there was a general trend for decrease in faecal output for all the groups. Unfortunately, the activities of animals were not evaluated for this study.

There were no changes in the haematological parameters attributable to administration of tested treatments. Albumin, triglycerides, and total cholesterol levels in β-sitosterol and chlorpropamide-treated mice were lower, relative to untreated-mice. Treatment of mice with doses of plant extract, β-sitosterol and chlorpropamide resulted in non-significant changes in total serum protein, globulin, alanine aminotransferase, alkaline phosphatase, urea nitrogen and creatinine. Necropsy morphological findings revealed that animals fed with plant extract showed large amounts of body internal fat including epicardial adipose, which, in recent studies, has been considered as an additional significant feature which may leads to diabetic myocardial complications [44–46]. The liver and kidneys for all treated and untreated groups appeared macroscopically normal and darker red pinpoint foci, mostly in the liver of some animals are likely associated with congestion of the organs. Most of the thymuses of the animals were small, which may relate to aging of animals or strain of mice [47, 48]. Small adrenal glands may indicate that all animals were not exposed to excessive chronic stress [49, 50], and specific histological changes could also not be detected in those adrenal glands that were microscopically evaluated.

There were some pathological signs in treated and untreated animals particularly in myocardial fibres, renal tubular, glomerular and hepatocyte granularity. Some of the lesions were more subacute and may be associated with hypoxia or cardiomyopathy [51]. The renal tubular changes in all the animal groups are likely hypoxic in origin thus likely terminal since the lesions are restricted to the deep cortical tissues where hypoxia would be firstly expected [27]. Interestingly, glomerular changes were visible with mesangial expansion in all animal groups. and mesangial expansion due to increase in mesangial matrix is described to occur in diabetic mice [52].

The animals in general did not demonstrate severe pathology associated with diabetes and thus can be considered a good model for early-stage diabetes. Moreover, there was a variation in the distribution pattern of endocrine islets of Langerhans. Morphometric analysis showed no clear indication of regeneration of the endocrine cells in pancreatic islets by the β-sitosterol. Thus, for further confirmation more immunofluorescence and tunel staining of the pancreas studies are recommended. Nonetheless, Prentki and Nolan [53], demonstrated degeneration in the pancreatic tissue of diabetic mice, vacuolization of Langerhan’s islet cells, decrease in the islets size, decrease in the β-cell numbers and also in the architecture of the islets. Loss of pancreatic β-cell mass and β-cell dysfunction is vital in the progress of type II diabetes and, in combination with peripheral insulin resistance which lead to hyperglycaemia. While β-cells fail to accurately secrete insulin at a provided glucose level, there is also a progressive decline in the number of β-cells [54]. In the present study the histopathologic changes of islets have been observed in the animals fed with Epol diet only and treated with the positive control drug showing some islets which were hyperplasia and others which were atrophy, while those treated with plant extract and compound showed hyperplasia. Since the pancreas responds to peripheral insulin resistance by increasing insulin production [55], the hyperplasia and absence of atrophy may be indicative of the antioxidant protective mentioned above.

An unexpected finding was the total increase in islet counts in the animals treated with β-sitosterol. This may be due to the fact that new islets do grow under certain experimental conditions, such as neogenesis, β-cell replication and differentiation [56, 57]. Collombat et al. [58] indicated that some plants induce regeneration of pancreatic islets in STZ-induced diabetic rats. However, there are many contradictory opinions and data surrounding these regeneration theories. The result in this study does tend to support some assertions [58].

## Conclusion

It can be concluded from this study that *H. hemerocallidae* is unlikely to be a suitable sole treatment agent in the management of prediabetes. Since none of the treatments could be considered highly effective for the management of type II pre-diabetes as sole therapeutic intervention. Moreover, the diabetic models of natural progress are crucial for investigating and developing novel drugs for diabetes and its complications.

## Supplementary Information

**Additional file 1.**

## Data Availability

All relevant data is contained in this manuscript.
