# Supplementary material for: Verification of the folkloric and anecdotal antidiabetic effects of Hypoxis hemerocallidea (Fisch., C.A. Mey. & Avé-Lall) and isolated, β-sitosterol using early-stage type II spontaneous diabetic mutant BKS-Leprdb mice
Source: BMC Complement Med Ther. 2022 Jun 20;22:163. doi: 10.1186/s12906-022-03640-y (PMC9208228; doi:10.1186/s12906-022-03640-y)
Supplement: Supplementary file 1 — Additional file 1. [file 12906_2022_3640_MOESM1_ESM.docx]

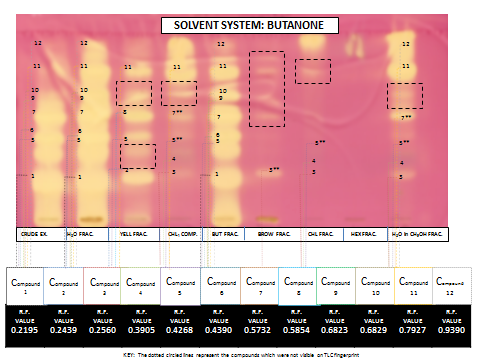

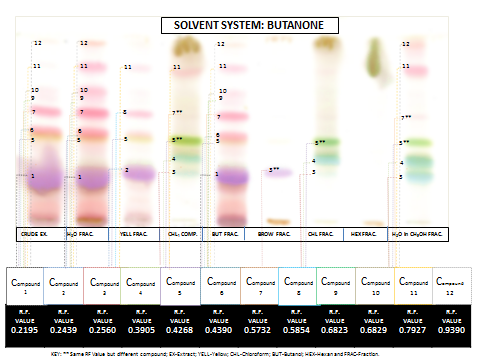


**SOLVENT SYSTEM: EBWF**

**SOLVENT SYSTEM: EBWF**

**Appendix 1.A.** Thin layer chromatography and anti-oxidant qualitative analysis of solvent fractionations using EBWF solvent system (A) sprayed with DPPH solution, (B) sprayed with vanillin in sulphuric acid. KEY: The dotted circled lines represent the compounds which were not visible on the TLC fingerprint sprayed with sprayed with vanillin in sulphuric acid; Rf: retention factor; ** : same Rf value; Ex: extract; Yell: yellow in colour; CHL: chloroform; BUT: Butanol; HEX: Hexane; Frac: fraction.


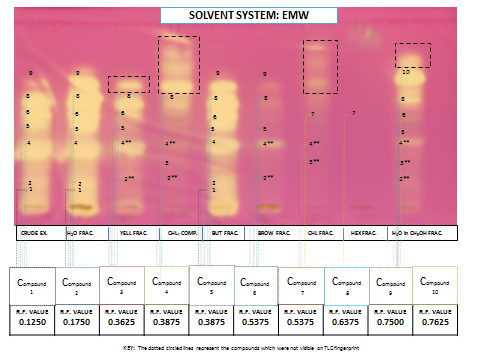

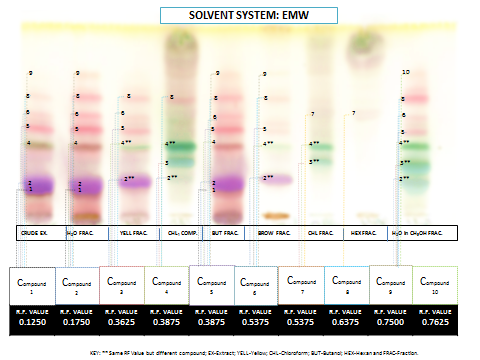


**Appendix 2.A.** Thin layer chromatography and anti-oxidant qualitative analysis of solvent fractionations using EMW solvent system (A) sprayed with DPPH solution, (B) sprayed with vanillin in sulphuric acid. KEY: The dotted circled lines represent the compounds which were not visible on the TLC fingerprint sprayed with sprayed with vanillin in sulphuric acid; Rf: retention factor; ** : same Rf value; Ex: extract; Yell: yellow in colour; CHL: chloroform; BUT: Butanol; HEX: Hexane; Frac: fraction.


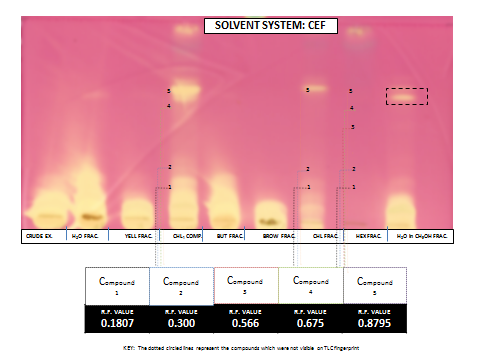

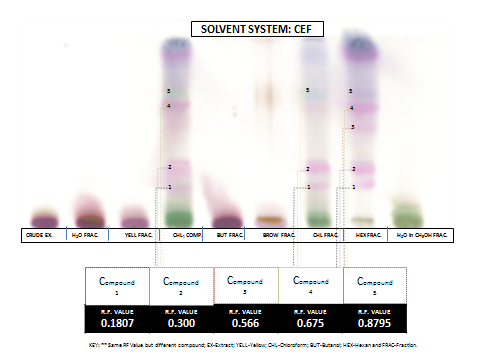


**Appendix 3.A.** Thin layer chromatography and anti-oxidant qualitative analysis of solvent fractionations using CEF solvent system (A) sprayed with DPPH solution, (B) sprayed with vanillin in sulphuric acid. KEY: The dotted circled lines represent the compounds which were not visible on the TLC fingerprint sprayed with vanillin in sulphuric acid; Rf: retention factor; **: same Rf value; Ex: extract; Yell: yellow in colour; CHL: chloroform; BUT: Butanol; HEX: Hexane; Frac: fraction.

**
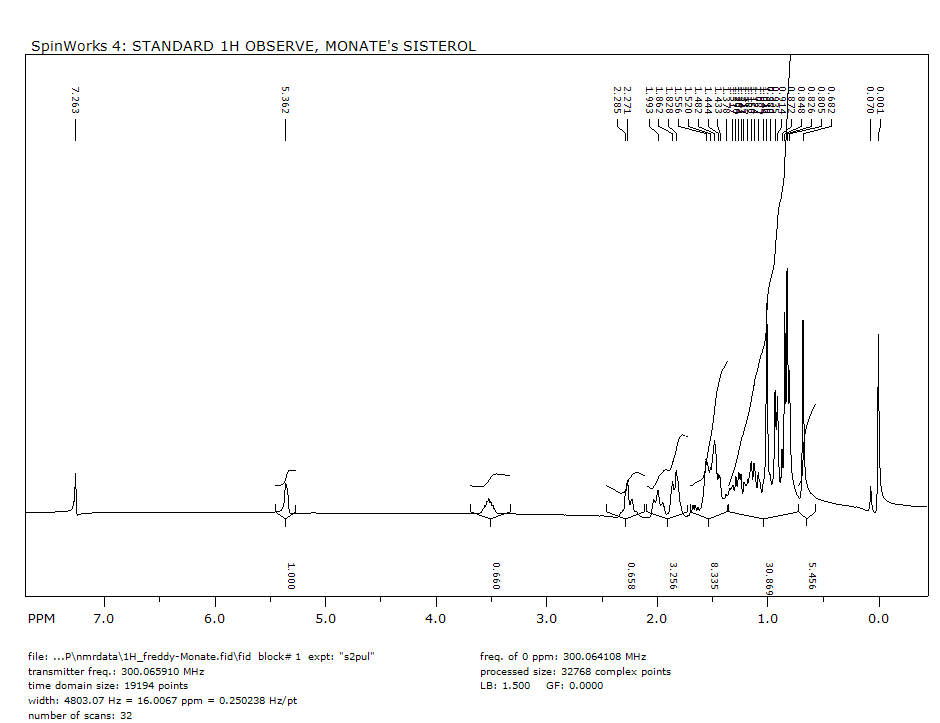
**

**Appendix 1.B.** β-sitosterol compound (coded PRCD): 13C.

**
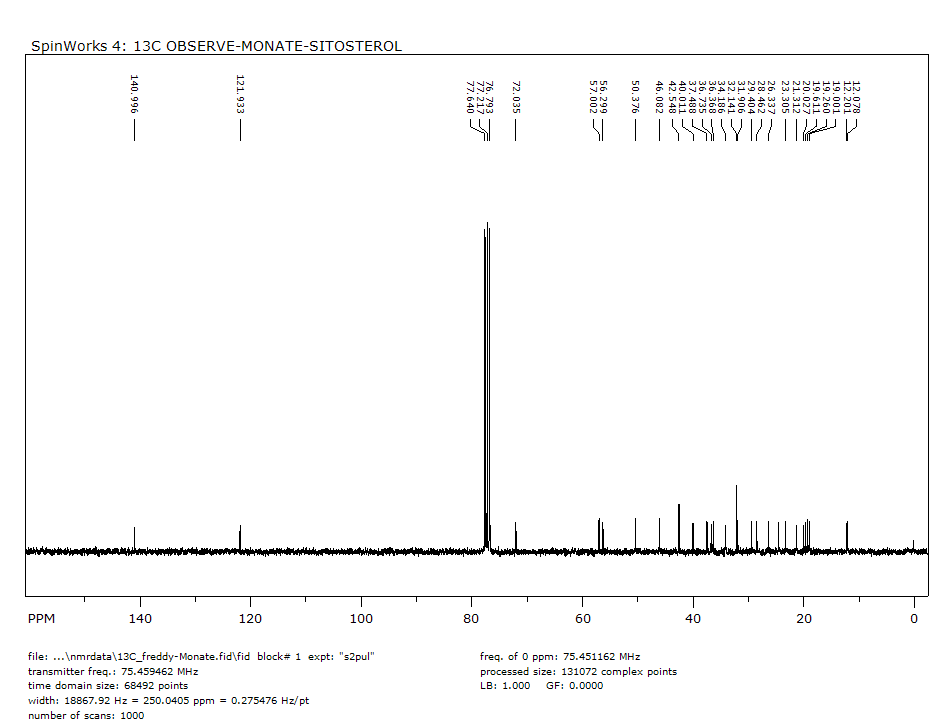
**

**Appendix 2.B.** β-sitosterol compound (coded PRCD): 1H-NMR spectrum.

**Appendix 3.B.** 1H-1H COSY spectrum of β-sitosterol compound.

**Appendix 4.B.** HMBC spectrum of β-sitosterol compound.

**Appendix 5.B.** HMQC spectrum of β-sitosterol compound.
